# Development of Serum-Free Culture Systems for an Immortalized Porcine Kidney-Derived Macrophage Cell Line

**DOI:** 10.3390/ani15040558

**Published:** 2025-02-14

**Authors:** Seiki Haraguchi, Takato Takenouchi, Kentaro Masujin, Shunichi Suzuki, Takehiro Kokuho, Hirohide Uenishi

**Affiliations:** 1 Division of Biomaterial Sciences, Institute of Agrobiological Sciences, National Agriculture and Food Research Organization, 2 Ikenodai, Tsukuba 305-0901, Japan; ttakenou@affrc.go.jp (T.T.); shunsuzu@affrc.go.jp (S.S.); huenishi@affrc.go.jp (H.U.); 2 Division of Transboundary Animal Disease Research, National Institute of Animal Health, National Agriculture and Food Research Organization, 6-20-1 Josuihoncho, Kodaira 187-0022, Japan; masujin@affrc.go.jp (K.M.); takehiro@affrc.go.jp (T.K.)

**Keywords:** immortalized porcine kidney-derived macrophage cell line, African swine fever virus, serum-free culture, suspension culture, polyvinylpyrrolidone K90

## Abstract

The immortalized porcine kidney-derived macrophage (IPKM) cell line is an excellent resource that has proven to be an effective for the production of various strains of African swine fever virus (ASFV). In this study, we developed a serum-free medium to replace the standard serum-containing medium and established the long-term subculture of IPKM cells. We used KnockOut serum replacement instead of serum. As the expression of several cytokines and their receptors were observed in IPKM cells, we examined their effect and found that porcine colony stimulating factor 1 (pCSF1) and pCSF2 were essential for the proliferation of IPKM cells. Furthermore, the addition of 2% polyvinylpyrrolidone K90 to the medium improved cell adhesion and allowed for the stable long-term subculture of IPKM cells. The proportion of cells with a normal number of chromosomes (2n = 38) was similar to that of IPKM cells cultured in a serum-containing medium. Moreover, we investigated the spinner flask suspension culture without using microcarrier beads. Finally, we developed a modified serum- and calcium-free medium in which IPKM cells could be successfully cultured for an extended period. Whether grown in adherent or suspension culture, the expression of the macrophage-specific markers, CD172a, CD203a, and Iba1, remained positive, indicating that IPKM cells retained their macrophage properties.

## Simple Summary

The immortalized porcine kidney-derived macrophage (IPKM) cell line that we established in 2017 has been demonstrated to be highly susceptible to cell-culture–adapted and field isolates of African swine fever virus (ASFV). The cells used to produce viruses for cell culture vaccines must be confirmed to be free from contamination of adventitious pathogen contamination. Therefore, it is desirable to establish a serum-free culture system to reduce the potential risk of unexpected contamination in final vaccine products. In this study, we successfully developed a novel serum-free medium, which enabled the long-term adherent and suspension subcultures of IPKM cells. Whether grown in an adherent or suspension culture, IPKM cells retained their original macrophage characteristics.

## 1. Introduction

The target cells of the African swine fever virus (ASFV) are known to be macrophages and monocytes [1,2,3]. Thus, immortalized cell lines of these should substantially contribute to the development of an ASFV vaccine. We previously established an immortalized porcine kidney-derived macrophage (IPKM) cell line by introducing the SV40 large T antigen and porcine telomerase reverse transcriptase genes into primary porcine kidney-derived macrophages using lentiviral vectors [4]. The IPKM cell line has been shown to support ASFV growth without the need for an initial adaptation period [5]. Furthermore, it is highly susceptible to both cell-cultured-adapted and field isolates of ASFV and is an excellent sustainable resource [5,6,7,8,9,10].

The development and production of vaccines depend on the successful cultivation of cells that act as hosts for viral replication. Fetal bovine serum (FBS) contains nutrients, hormones, growth, and attachment factors and is highly effective in promoting cell growth and enhancing viral replication [11]. However, the use of serum has some disadvantages, including high cost, variability in performance between batches, and the potential to contain unwanted contaminants, which are major safety concerns for biological products. Furthermore, there are ethical considerations regarding killing unborn animals. This issue assumes particular significance in the context of the principle of the 3Rs (Replacement, Reduction, and Refinement). Given these drawbacks, the use of serum-free media is desirable. There have been reports of the production of influenza vaccines using serum-free cultured MDCK cells [12,13,14,15], the propagation of the foot-and-mouth disease virus in BHK cells [16,17], and the proliferation of the canine adenovirus type 2 in MDCK cells [18]. Generally, normal diploid adherent cells are anchorage-dependent and have difficulty adapting to a suspension culture, which often necessitates the use of scaffold materials [19]. Microcarrier beads have been used as a scaffold in suspension culture systems [12,13,14,15,16,17,18].

In the present study, we report the development of serum-free media and their use in the adherent and suspension cultures of IPKM cells. First, based on our cell culture experience, we used KnockOut Serum Replacement (KSR) as a serum replacement [20]. Second, we evaluated cell growth while adding various additives, such as cytokines and polymer compounds, to the medium and ultimately developed a serum-free medium that allowed for the long-term adherent subculture of IPKM cells. Lastly, we explored a suspension culture method for IPKM cells using a serum-free medium. As mentioned above, the use of scaffold materials is considered necessary for a suspension culture; therefore, we aimed to develop a microcarrier-free suspension culture method with an emphasis on simplicity. While improving the serum-free medium to prevent cells from attaching to the devices, we succeeded in culturing IPKM cells in suspension using spinner flasks. From this perspective, these findings are expected to lead to the future development of live-attenuated vaccines that proliferate in IPKM cell vaccines using serum-free IPKM cell cultures.

## 2. Materials and Methods

### 2.1. General Procedures for Cell Culture and Media Preparation

*Culture media and supplements*: All media used in this study are summarized in Table 1 and the manufacturers of each reagent are listed below: Dulbecco’s Modified Eagle Medium (DMEM) (#08458-45, Nacalai Tesque, Inc., Kyoto, Japan), alpha Modified Eagle Minimum Essential Medium (α-MEM) (#21444-05, Nacalai Tesque), calcium-free DMEM (#16972-45, Nacalai Tesque), monothioglycerol (MTG) (FUJIFILM Wako Pure Chemical Corporation, Osaka, Japan), antibiotic-antimycotic mixed stock solution (Anti-Anti) (Nacalai Tesque), FBS (CCP-FBS-BR-500, COSMO BIO Co., Ltd., Tokyo, Japan), KSR (Thermo Fisher Scientific Inc., Waltham, MA, USA), GlutaMAX Supplement (Thermo Fisher Scientific), sodium pyruvate solution (Nacalai Tesque), EmbryoMax nucleosides (Sigma-Aldrich, Co., St. Louis, MO, USA), insulin (I0516, Sigma-Aldrich), polyvinyl alcohol (PVA) (P8136, #363081, #363146, all from Sigma-Aldrich), polyvinylpyrrolidone K90 (PVP K90) (#28354-04, Nacalai Tesque), and Dulbecco’s phosphate-buffered saline without calcium and magnesium (D-PBS(−)) (Nacalai Tesque).

*Serum-containing standard medium (FBSstd)*: As previously reported [4], the original culture medium for IPKM cells was DMEM containing 10% heat-inactivated FBS supplemented with 25 μM MTG, 10 μg/mL insulin, and 1% Anti-Anti solution (Table 1).

*Serum-free media*: The composition of each of the serum-free KSR-based media is presented in Table 1, where the media were designated as DKSR + K90, αKSR + K90, or Ca(−)KSR + K90, depending on the type of medium and the presence or absence of PVP K90.

*Preparation of the cells*: IPKM cells frozen in CELLBANKER1 (ZENOGEN PHARMA, Fukushima, Japan) were thawed in 5 mL of DMEM containing 10% FBS. After centrifugation, the supernatant was removed, and the cells were washed twice with 5 mL of D-PBS(−) before starting the cell culture. All cell cultures were performed in humidified 5% CO_2_ air at 38 °C. The day of cell inoculation was designated as day 0.

*Adherent cell culture and passage*: IPKM cells (2.5 × 10^5^) were cultured on 35 mm nontissue culture-grade plastic dishes (MS-1135R, Sumitomo Bakelite Co., Ltd., Tokyo, Japan). The medium was changed every 3–4 days. TrypLE Express Enzyme (Thermo Fisher Scientific) was used to detach the cells for passaging. Cells were counted using a TC20 Automated Cell Counter (Bio-Rad Laboratories, Inc., Hercules, CA, USA), and cell viability was assessed via Trypan Blue (Sigma-Aldrich) staining when necessary.

*Suspension cell culture using dishes*: To prevent cells from attaching to the surface, the 35 mm dishes were coated with 0.5 mL of 0.3% Poly(HEMA) (P3932, Sigma-Aldrich), as previously described [21]. After the Poly(HEMA) solution had completely dried, 2.5 × 10^5^ IPKM cells were seeded in 3 mL of medium and cultured for up to 7 days and then evaluated.

*Suspension cell culture using Erlenmeyer flasks*: The insides of 50 mL Erlenmeyer glass flasks (#4980FK50, AGC TECGNO GLASS Co., Ltd., Shizuka, Japan) were treated with Sigmacote (SL2, Sigma-Aldrich). Thereafter, 1 × 10^6^ IPKM cells were inoculated into 25 mL of medium. The flasks were placed on an orbital shaker (OS-762, OPTIMA Inc., Tokyo, Japan) installed in a CO_2_ incubator, and the cells were incubated with shaking at 100 rpm for up to 7 days and then evaluated.

*Suspension cell culture using spinner flasks*: We used 125 mL and 500 mL spinner flasks (#3152 and #3153, respectively, Corning, NY, USA) and a slow stirrer device (ULS-4N, AS ONE Corporation, Osaka, Japan) in a CO_2_ incubator. In the experiments, to determine the optimal spinner speed and culture conditions, 3 × 10^6^ IPKM cells were placed in 30 mL of medium and cultured for 10 days in 125 mL spinner flasks, after which the proliferation rate was evaluated. For the long-term suspension culture, 3 × 10^6^ and 6 × 10^6^ IPKM cells were each suspended in 30 mL of Ca(−)KSR + K90 and cultured in a 125 mL spinner flask at 50 rpm (day 0) to compare the proliferation efficiency. Every 4 days, 2 mL of the medium was harvested, and the cells were counted after dispersing the cell clumps using Accumax (Nacalai Tesque), and then 12 mL of fresh medium was added. On day 20 (total medium 70 mL), all cells were collected by centrifugation at 280× *g* for 5 min. After removing the supernatant, the cells were suspended in 100 mL of fresh Ca(−)KSR + K90 by gentle pipetting and cultured in a 500 mL spinner flask at 60 rpm. Similarly, cells were counted, and 52 mL of fresh medium was added every 4 days with gentle pipetting. The suspension culture continued until day 40 (total medium 300 mL).

### 2.2. Preparation of pCSF1 and pCSF2 Recombinant Proteins

The cloning of genes and biological assay were performed as previously described for pLIF [22]. Briefly, cDNAs of pCSF1 (synonym for macrophage colony-stimulating factor, NM_001244523.1) and pCSF2 (synonym for granulocyte-macrophage colony-stimulating factor, NM_214118.2) were prepared from total RNA extracted from porcine blastocysts, cloned into a pCAGGS vector, and sequenced. The primers used are listed in Supplemental Table S1. The purified plasmid was electroporated into COS-7 cells. After 24 h of culturing, the cells were washed with D-PBS(−) and cultured in Opti-MEM I Reduced Serum Medium (Thermo Fisher Scientific) for 3 days. The supernatants were recovered, concentrated using Vivaspin 15R, VS15RH12 (Sartorius Stedim Lab Ltd., Stonehouse, UK), and exchanged with D-PBS(−). For the biological assay, 2 × 10^5^ IPKM cells were plated onto 35 mm dishes with DMEM containing 15% KSR, 25 μM MTG, and 1% Anti-Anti solution, and each recombinant protein was added to the medium in a 10-fold serial dilution. Concentrations that were effective for cell proliferation were used. Typically, a 400-fold dilution (0.25%) was sufficient.

### 2.3. Evaluation of Cytokines and Polymer Compounds on Cell Proliferation

The serum-free medium used to evaluate the effects of cytokines consisted of DMEM containing 15% KSR, 25 μM MTG, and 1% Anti-Anti. In addition, pLIF, pCSF1, and pCSF2 were each added to the medium at a 400-fold dilution (0.25%). hIL-4 (A42603, Thermo Fisher Scientific) was used at a concentration of 20 ng/mL. The medium used to examine the effects of the polymer compounds consisted of DMEM containing 15% KSR, 25 μM MTG, 1% Anti-Anti, 0.25% pCSF1, and 0.25% pCSF2. Each of the three types of PVA (P8136, #363081, #363146) were dissolved in distilled water at a 10% (*w*/*v*) concentration, autoclaved, and added to the medium at a final concentration of 0.1%. PVP K90 was added to the medium at a concentration of 2.0% (*w*/*v*), dissolved at 4 °C with gentle shaking, filtered through a 0.2-μm membrane, and diluted as necessary. Thereafter, 2.5 × 10^5^ IPKM cells were plated onto 35 mm dishes with medium containing either each cytokine, 0.1% PVA, or 0.1–2.0% PVP K90. The cells were then split 1:2 after 3–4 days of culture. Three days after 3 passages, the cells were counted and the growth rates were evaluated.

### 2.4. Cell Proliferation Assay

Cell growth rate was determined based on population doubling calculated by using the following formula:

log (cells harvested/cells seeded)/log2

**Figure 1:**
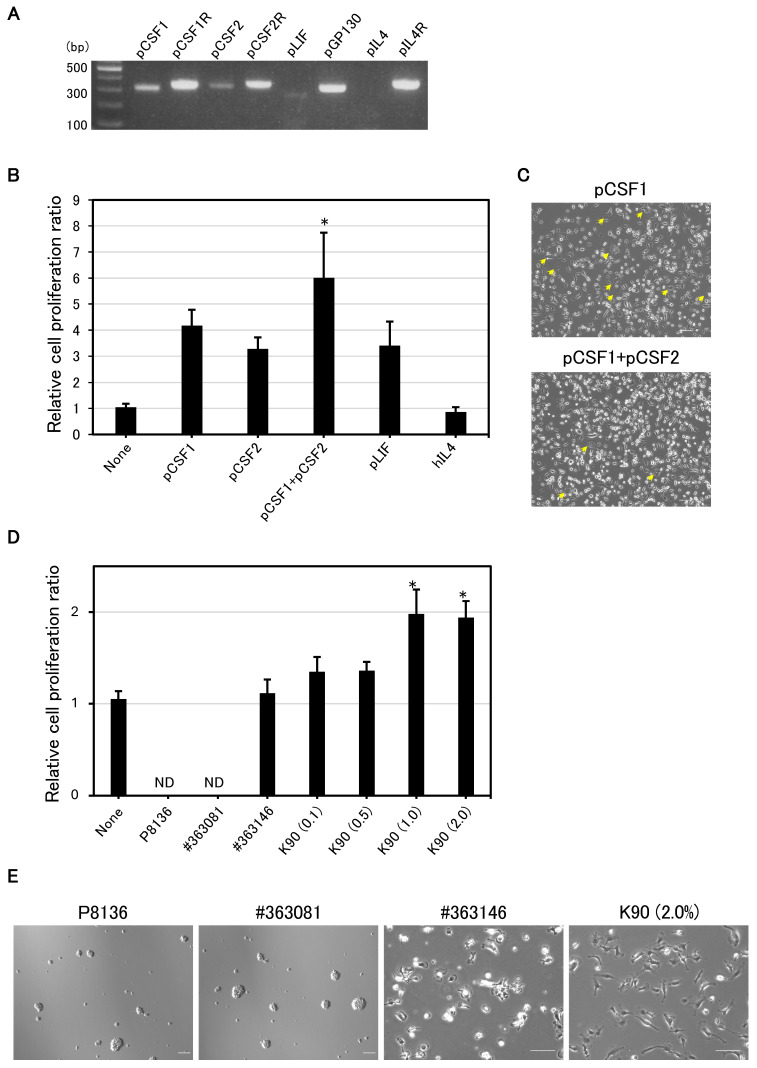
Effects of cytokines and hydrophilic polymers on the serum-free adherent culture of IPKM cells. (**A**) mRNA expression status of cytokines and their receptors in IPKM cells. The cytokine expression was highest in the order of pCSF1, pCSF2, and pLIF. The expression of pIL4 was not detected. The expression of all cytokine receptors (pCSF1R, pCSF2R, pGP130, and pIL4R) was high compared with that of the cytokines. Note that PCR was performed for 38 cycles, except for pLIF and pIL4, for which it was performed for 40 cycles. (**B**) All cytokines except hIL4 induced cell proliferation. The proliferation rate was significantly higher than the control (None) when pCSF1 and pCSF2 were both added to the medium (Dunnett test, * *p* = 0.002). The addition of hIL4 had no effect on the proliferation rate and caused cell death. Experiments were repeated four times. (**C**) When pCSF1 was added alone, it caused frequent syncytia (arrows), but the simultaneous addition of pCSF1 and pCSF2 reduced the incidence thereof. Note that the cells are weakly attached. Results are shown after two passages. (**D**,**E**) Polyvinyl alcohol (PVA), such as P8136, #363081, and #363146, had no effect on cell growth. Cells cultured in medium with added P8136 and #363081 did not adhere to the dish and formed clusters. PVP K90 in the medium exhibited a significant increase in the cell proliferation rate at concentrations of 1.0% and 2.0% compared with the control (None) (Dunnett test, * *p* < 0.001). The most stable cell attachment was observed at 2.0% concentration. The experiments were repeated four times. None—medium without added cytokines; ND—not determined due to the extremely small cell number; all scale bars = 100 μm.

**Figure 2:**
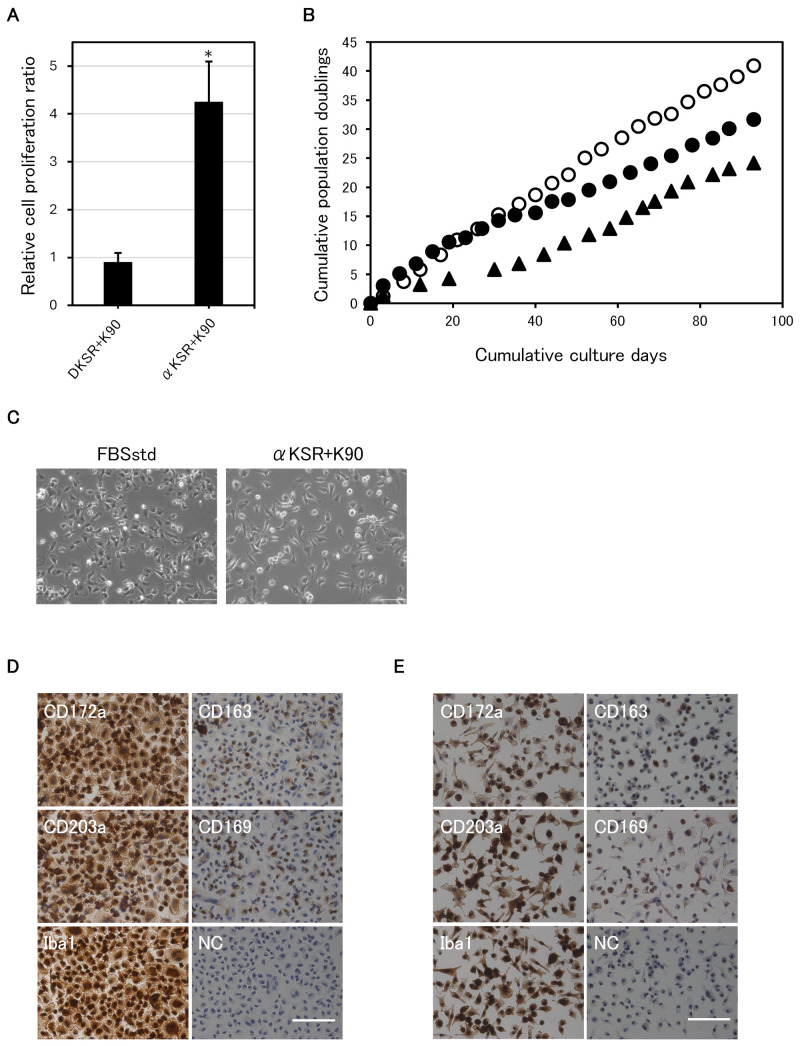
Successful long-term serum-free adherent subculture of IPKM cells. (**A**) The rate of IPKM cell proliferation was approximately four times higher in αMEM (αKSR + K90) than in DMEM (DKSR + K90) (* *p* = 0.0072). (**B**) The cumulative population doublings of IPKM were plotted against the duration of the culture period (days). Two independent passage culture experiments provided similar proliferation curves. Open circles—IPKM cells cultured in serum-containing medium (FBSstd); closed circles—IPKM cells cultured in serum-free and PVP-containing medium (αKSR + K90); and closed triangles—IPKM cells cultured in serum- and PVP-free medium (αKSR−K90). The composition of each media is presented in Table 1. (**C**) Morphology of IPKM cells subcultured in FBSstd and αKSR + K90 for 3 months. (**D**,**E**) Expression of macrophage-specific markers after long-term culture. The marker expression in IPKM cells cultured in αKSR + K90 (**E**) was consistent with that of cells cultured in FBSstd (**D**). The macrophage-specific markers CD172a, CD203a, and Iba1 remained positive, and CD163a and CD169 were positive in some cells. NC—negative control using secondary antibody only; all scale bars = 100 μm.

**Figure 3:**
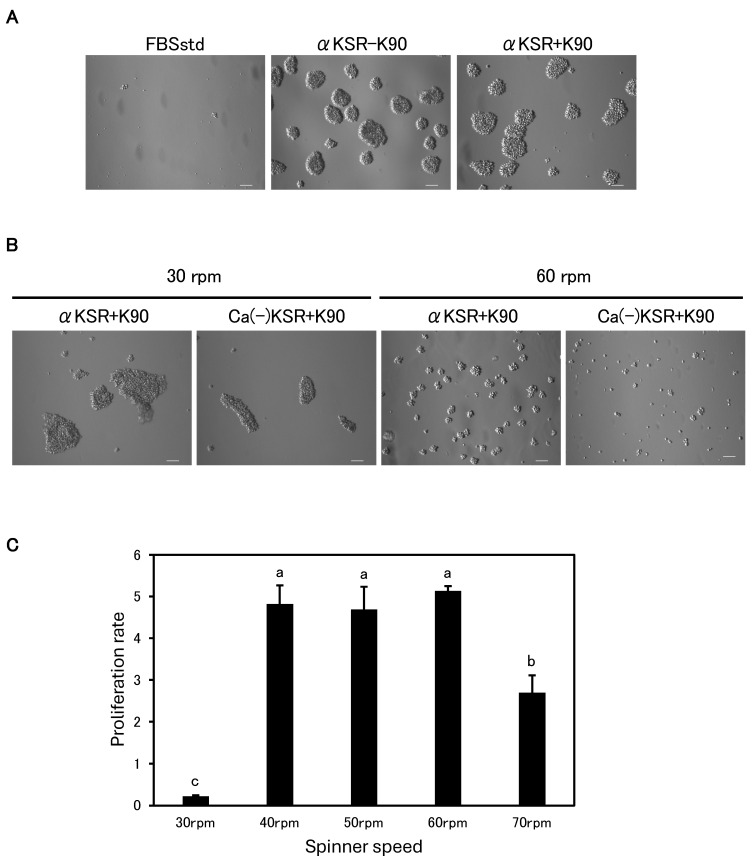
Optimal media and spinner parameters for the suspension culture of IPKM cells. (**A**) Suspension culture in poly(HEMA)-coated 35 mm dishes. IPKM cells cultured in FBSstd did not proliferate. In αKSR−K90 and αKSR + K90, the cells formed aggregates and became large clusters by day 7. (**B**) Differences in cluster formation when cultured in spinner flasks. Comparison between the cells cultured in αKSR + K90 and Ca(−)KSR + K90, and between cells cultured at 30 and 60 rpm. Cells cultivated in Ca(−)KSR + K90 at a spinner speed of 40–60 rpm provided the optimal conditions to reduce clump size. (**C**) Cell proliferation rate at different spinner speeds. IPKM cells were cultured in Ca(−)KSR + K90. Different letters (a–c) indicate significant differences (Tukey–Kramer test, *p* < 0.01). The experiments were repeated three times. All scale bars = 100 μm.

**Figure 4:**
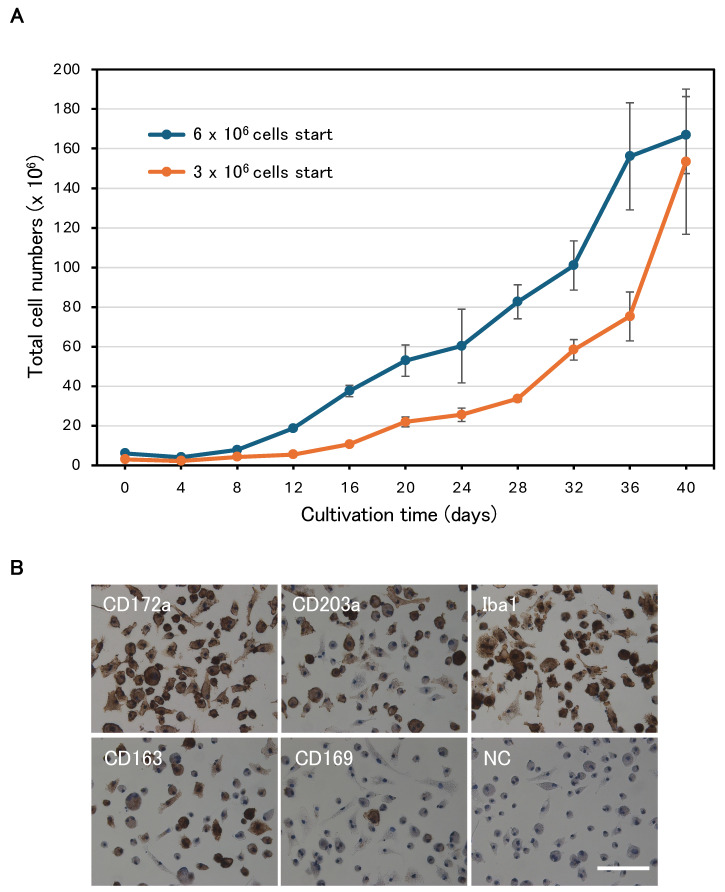
Successful long-term serum-free suspension culture of IPKM cells. (**A**) IPKM cells were cultured at a density of 3 × 10^6^ and 6 × 10^6^ in 30 mL of Ca(−)KSR + K90 using 125 mL spinner flasks (50 rpm). Every 4 days, 2 mL of the medium was collected to count the cells, and then 12 mL of fresh medium was added. On day 20 (total 70 mL), the cells were then transferred to 500 mL spinner flasks (60 rpm). Thereafter, every 4 days the cells were counted, and 52 mL of fresh medium was added in the same manner until day 40 (total 300 mL). Experiments were repeated three times. (**B**) Expression of macrophage-specific markers after long-term culture. Scale bar = 100 μm.

Relative cell proliferation ratios for cytokines (Figure 1B), polymers (Figure 1D), and DMEM vs. αMEM (Figure 2A) were determined on day 3, after three passages, and expressed as the mean ± SD (n = 4). Cumulative population doubling (Figure 2B) was calculated by summing the population doublings in culture over time. For evaluating the optimal spinner speed (Figure 3C), total cell numbers were counted after 10 days of culture and the mean ± SD (n = 4) was plotted. For long-term serum-free suspension culture (Figure 4A), total cell numbers were counted every 4 days and the mean ± SD (n = 3) was plotted. 

### 2.5. RT-PCR

The details of this process have been previously described [20,22]. In brief, total RNA from IPKM cells was prepared using an RNeasy Plus Mini Kit (QIAGEN GmbH, Hilden, Germany). First-strand cDNA was synthesized using a PrimeScript II 1st Strand cDNA Synthesis Kit (Takara Bio Inc., Shiga, Japan), and PCR was performed with EmeraldAmp MAX PCR Master Mix (Takara Bio) with the following conditions: 38 (for pCSF1, pCSF2, and all receptors) or 40 (for pLIF and pIL4) cycles at 98 °C for 10 s and 68 °C for 40 s. The PCR primers and product sizes are listed in Supplemental Table S2.

### 2.6. Immunocytochemistry

The procedure was performed following a previously reported method [4]. IPKM cells were seeded in eight-well chamber slides (Asahi Glass Co., Ltd., Tokyo, Japan) at a density of 1.5 × 10^5^ cells/well. After being washed once with D-PBS(−), the cells were fixed with 4% paraformaldehyde in PBS, permeabilized with 1% Triton X-100 in PBS, and blocked in Blocking One Histo buffer (Nacalai Tesque). The cells were then incubated with the primary antibodies for 1 h at room temperature, and the EnVision system (DAKO, Hamburg, Germany) was used to visualize the antibody–antigen reactions, according to the manufacturer’s procedure. The cell nuclei were counterstained with Mayer’s hematoxylin solution (FUJIFILM Wako), and the stained slides were examined under a microscope. The primary antibodies used for immunocytochemistry were as follows: mouse monoclonal antibodies against CD163 (MCA2311, Bio-Rad), CD169 (MCA2316, Bio-Rad), CD172a (VMRD, Inc., Pullman, WA, USA), and CD203a (MCA1973, Bio-Rad) and rabbit polyclonal antibodies against ionized calcium-binding adaptor molecule 1 (Iba1) (#019-19741, FUJIFILM Wako).

### 2.7. Chromosome Number Analysis

IPKM cells were incubated with a medium containing 60 ng/mL of demecolcine (FUJIFILM Wako) for 6 h at 38 °C in a 5% CO_2_ incubator. After trypsinization, the cells were gently resuspended in 5 mL of 75 mM KCl and incubated at 37 °C for 15 min. Thereafter, the cells were fixed by the addition of 0.5 mL of methanol/glacial acetic acid solution (3:1). The cells were pelleted and resuspended in 5 mL of fresh fixative solution. The samples were placed dropwise onto slides to prepare chromosome spreads, and the dried slides were stained with Leishman solution. Approximately 50 metaphase spreads were counted for each sample.

### 2.8. Statistical Analysis

Statistical analysis was performed using KyPlot 6.0 (KyensLab Inc., Tokyo, Japan). The Tukey test was used for multiple comparisons, and comparisons with the control group were performed using the Dunnett test. Student’s *t*-test was used for paired data. A *p*-value of less than 0.05 (*p* < 0.05) was considered statistically significant.

## 3. Results

### 3.1. pCSF1 and pCSF2 Support the Proliferation of IPKM Cells

We first examined the expression of several major cytokines and their receptors in IPKM cells. Of the four cytokines examined, pCSF1 had the highest relative expression, followed by pCSF2 and pLIF (Figure 1A), whereas pIL4 expression was not detected even after 40 reaction cycles. Because these receptors (pCSF1R, pCSF2R, pGP130, and pIL4R) exhibited a relatively high expression (Figure 1A), we added each of these cytokines to the serum-free medium to determine which of them could support cell proliferation. The IPKM cells were unable to grow without the cytokine(s) (Figure 1B). Consistent with the cytokine mRNA expression levels, pCSF1, pCSF2, and pLIF were effective in promoting cell proliferation over 2–3 passages, whereas hIL4 had no proliferative effect but rather induced cell death (Figure 1B). When IPKM cells were subcultured in pCSF1-supplemented medium, syncytium-like multinucleate large cells were frequently observed (arrows in Figure 1C). However, when pCSF1 and pCSF2 were simultaneously added to the serum-free medium, the appearance of these syncytium-like cells was reduced, and the cell proliferation rate was significantly higher than the control without cytokine (*p* = 0.002, Figure 1B,C). It was found that the addition of pCSF1 and pCSF2 supported IPKM cell growth, but the cells in this medium tended to detach easily from the dish (Figure 1C).

### 3.2. PVP K90 Addition Improves Cell Growth and Adhesion

To address the concerns about cell growth and adhesion, we investigated the effect of the water-soluble polymer compounds PVA (P8136, #363081, and #363146) and PVP K90. Compared to the control without polymer, none of the PVAs were effective (Figure 1D). When cells were cultured in a medium supplemented with P8136 and #363081, they did not adhere to the dish but instead formed clumps and did not proliferate; thus, the number of cells was extremely low and could not be counted (Figure 1D,E). Meanwhile, cells cultured in a medium supplemented with PVP K90 exhibited an increase in proliferation and adhesion rates (Figure 1D,E), with higher proliferation rates at 1.0% and 2.0% (*p* < 0.001). Empirically, 2.0% PVP K90 had better cell attachment efficiency compared with 1.0% PVP in the subculture process; the concentration was fixed at 2.0% for subsequent experiments. 

### 3.3. α-MEM Is Superior to DMEM for the Serum-Free Culture of IPKM Cells

Based on our experience, we hypothesized that the proliferation efficiency could be further improved by using α-MEM (αKSR + K90) instead of DMEM (DKSR + K90) (Table 1). As expected, after two passages, IPKM cells cultured in αKSR + K90 exhibited a 4-fold higher cell proliferation rate than cells cultured in DKSR + K90 (*p* = 0.0072) (Figure 2A). From these results, we used αKSR + K90 as the serum-free medium for subsequent adherent cell cultures.

### 3.4. Successful Long-Term Serum-Free Adherent Subculture

We performed a long-term passaging culture of IPKM cells using αKSR + K90. As a control, we compared it with FBSstd and αKSR−K90 (without PVP K90) to confirm the effect of PVP K90 on the long-term passaging cultures. As shown in Figure 2B, we successfully cultured IPKM cells for more than 90 days in our serum-free culture system, and the results were similar in two independent experiments. The passage numbers were P22 (FBSstd), P21 (αKSR + K90), and P16 (αKSR−K90). Although the total cell numbers were higher in FBSstd than in αKSR + K90, IPKM cells cultured in αKSR + K90 had higher proliferation rates than those cultured in FBSstd for the first six passages. The growth rate of cells cultured in αKSR−K90 was slower than that of cells cultured in the other two media, thus indicating the effectiveness of PVP K90. For IPKM cells cultured for an extended period in αKSR + K90, their morphology was elongated, which was unlike that of the IPKM cells cultured in FBSstd (Figure 2C); however, they proliferated stably throughout the subculture period. The expression of the macrophage-specific markers CD172a, CD203a, and Iba1 in IPKM cells cultured in FBSstd for 93 days (P22) is shown in Figure 2D. In the original IPKM cells, the CD163 and CD169 expression patterns exhibited a mixture of positive and negative cells (Figure 2D). Consistent with these results, IPKM cells cultured with αKSR + K90 for 93 days (P21) maintained the expression status of macrophage-specific markers of the original IPKM cells (Figure 2E). The proportion of normal chromosomal numbers (2n = 38) was almost the same as that of the FBSstd control (64.3% vs. 65.3%) (Table 2). These results indicate that IPKM cells cultured in αKSR + K90 retain their initial characteristics.

### 3.5. Successful Long-Term Serum-Free Suspension Subculture

Next, we investigated whether IPKM cells could grow in a serum-free suspension culture. First, we examined whether the cells could grow while floating freely in the dishes. The dishes were pretreated with poly(HEMA) to prevent the cells from attaching to the surface. IPKM cells cultured in FBSstd did not proliferate but rather underwent cell death (Figure 3A). In contrast, cells cultured in αKSR−K90 and αKSR + K90 formed aggregates on day 1, and by day 7 these had become larger clumps (Figure 3A). To reduce aggregation and facilitate cell growth, we performed suspension cultures using Erlenmeyer glass flasks with constant agitation. The shaking action prevented cell clumping to an extent, and the cells were evenly distributed throughout αKSR + K90. Despite the poly(HEMA)-coated surface, many cells were observed to be attached, particularly to the bottom corners of the flasks. We discontinued the suspension cultures using Erlenmeyer flasks because increasing the shaker speed to prevent cell adhesion increased cell death. To identify better suspension culture conditions, we explored the potential of spinner flasks. The cells cultured in αKSR + K90 were able to proliferate, and the size of the clumps was reduced; however, the cells still adhered to the surface. Eventually, we opted to use a Ca-free medium with an increased KSR concentration of 20% (Ca(−)KSR + K90) (Table 1). As a result, the clumps became smaller in size, and the cell grew without attaching to the surface (Figure 3B). We then investigated the optimal spinner speed while avoiding damage to the cells from shear stress. When the spinner speed was as low as 30 rpm, the cells formed large clusters, and little proliferation was observed (Figure 3B,C). Conversely, when the spinner speed was increased to 70 rpm, the cells died due to shear stress (Figure 3C), and a large amount of cellular debris was observed in the medium. The results revealed that 40–60 rpm was the optimal speed for the spinner flask culture of IPKM cells (*p* < 0.01), and the cells were spherical and generally grew in the suspension without attaching to the surface. Unexpectedly, IPKM cells did not grow in the serum-containing medium and died even when using spinner flasks. Finally, we examined whether the long-term suspension culture of IPKM cells was possible using this spinner culture system. In contrast to the adherent culture, the cell number remained almost the same or slightly decreased for approximately 1 week after culture initiation, indicating a lag phase. The cells then entered the log phase and steadily increased and reached a plateau phase around day 40 (Figure 4A). Although the proliferation rate of the culture with 6 × 10^6^ cells at the beginning was higher than that with the 3 × 10^6^ cells, there was no significant difference in the total cell number after 40 days of culture (1.67 ± 19.4 × 10^8^ vs. 1.53 ± 36.7 × 10^8^, respectively). As with the adherent culture in a serum-free medium, the expression of CD172a, CD203a, and Iba1 was positive, and the expression pattern of CD163 and CD169 was a mixture of positive and negative cells at 40 days of culture (Figure 4B). Thus, IPKM cells maintained their macrophage characteristics after long-term suspension culture.

## 4. Discussion

To establish a serum-free culture system for IPKM cells, we first replaced 10% FBS with 15% KSR. We predicted that simply replacing FBS with KSR would not be sufficient for the subculture of IPKM cells and that the addition of some kind of factor, such as cytokines, would be necessary. Although there are many types of cytokines involved in the proliferation and differentiation of macrophages [23,24,25], we specifically focused on CSF1, CSF2, LIF, and IL4 and examined the expression status of these cytokines and their receptors in IPKM cells. The expression levels of these cytokines were consistent with the effect of the cytokines in promoting IPKM cell proliferation, implying that IPKM cells may have an autocrine/paracrine signaling system that controls cell proliferation. IPKM cells did not proliferate in the medium without added cytokines. This indicates that IPKM cells require cytokines in a serum-free culture and, from the results of this study, that at least pCSF1 and pCSF2 support cell growth.

IPKM cells were originally established as a cell line that adhered to Petri dishes [4]. Furthermore, they have the unique property of not growing on surface-treated cell culture dishes or gelatin-coated dishes (unpublished data). Although the addition of pCSF1 and pCSF2 to the culture medium promoted IPKM cell proliferation, the cells tended to detach easily regardless of the type of culture dish used. Therefore, we investigated the possibility of using versatile synthetic polymers, such as PVA and PVP, to improve the cell adhesion to dishes as well as the proliferation rate. P8136, 87–90% hydrolyzed PVA, has previously been used as a chemically defined serum-free medium for the culture of mouse embryonic stem cells [26], bovine embryos [27], and porcine embryos [28]. In addition, #363081, 87–89% hydrolyzed PVA, and #363146, 99% hydrolyzed PVA, have been shown to effectively replace serum albumin for the ex vivo expansion of mouse hematopoietic stem cells [29]. However, these PVAs did not affect the adhesion and proliferation of IPKM cells. Meanwhile, PVP K90 with a molecular weight of 360,000 Da improved both cell attachment and proliferation at a concentration of 1–2%, especially in the 2% PVP. Previous studies have shown that the macromolecular crowded culture medium formed by the addition of PVP positively affects the growth, viability, and development of mouse [30] and bovine [31] oocytes. Although the role of PVP in IPKM cell cultures is unclear, we speculate that it may also be due to the macromolecular crowding effect [32,33]. Based on our experience, we expected that changing the medium from DMEM (DKSR + K90) to αMEM (αKSR + K90) containing ribonucleosides and deoxyribonucleosides would further increase the proliferation rate. The cell proliferation rate after two passages in αMEM was more than four times higher than that in DMEM. Similar results would be expected by adding nucleic acids to DMEM (see below), but regardless, we demonstrated the long-term passaging culture of IPKM cells with αKSR + K90. In our serum-free culture system, we did not perform any procedure to adapt the cells to a serum-free environment, and the cells were used immediately after thawing from cryopreservation. This may be the reason that IPKM cells cultured in αKSR + K90 exhibited a higher proliferation rate for the first six passages and then adapted to the serum-free environment and proliferated more slowly than the cells cultured in FBSstd. In the long-term subculture, the proliferation rate of cells cultured in αKSR + K90 was lower than that in FBSstd. There may be room for further improvement, such as the addition of some other supplement(s) to the serum-free medium, to achieve a cell growth rate comparable to or better than that of the serum-containing medium. Importantly, there was no change in the expression of macrophage-specific markers or the proportion of normal chromosome numbers during the long-term subculture in the serum-free medium that we developed, and the IPKM cells’ macrophage characteristics were maintained.

We succeeded in culturing adherent IPKM cells in the serum-free medium. Thus, we next explored the establishment of a suspension culture system for large-scale and long-term cell culture. First, we tried a simple suspension culture using poly(HEMA)-treated dishes, but the cells aggregated and formed clumps that gradually grew like snowballs and did not proliferate. Eventually, we considered that the IPKM cell suspension cultures may require agitation to keep the cells in suspension and dispersed. Spinner flasks with a magnetic stirrer specifically designed for cell culture suspension allow for superior gas exchange and permit a higher volume of cells to be cultured. Using spinner flasks, we observed that IPKM cells in αKSR + K90 medium grew while forming small clumps, but the cells attached to the surface of the flask, including the paddles. The spinner parameters were critical, as increasing the spinner speed effectively inhibited cluster formation and cell adhesion to surfaces, but higher speeds also induced cell death due to shear stress. To avoid the attachment of the cells to surfaces while maintaining the proliferation efficiency, we attempted to use a Ca-free DMEM. As the cell proliferation rate in the adherent cell culture was found to be higher in αMEM (αKSR + K90) than in DMEM (DKSR + K90), we added glutamine, pyruvate, and nucleosides to make the composition of Ca(−)KSR + K90 closer to that of αKSR + K90. The results were encouraging: no cells were attached to the surface, clump sizes were smaller, and there was no reduction in the cell proliferation rate. We do not know whether Ca(−)KSR + K90 is completely free of calcium because the composition of KSR is not disclosed by the supplier. Even if KSR contains calcium, this calcium concentration is likely to have little effect on cell adhesion and aggregation. Various additives, such as PVA, PVP, dextran, and polyethylene glycol, have been used to protect freely suspended animal cells in culture from agitation [34,35]. Therefore, we examined the effects of the presence or absence of some of these polymers. The addition of PVAs, such as P8136, #363081, and #363146 to the medium, did not result in an improvement in the IPKM suspension cultures. An increase in efficacy was only observed with the addition of PVP K90. When the medium did not contain PVP K90 (Ca(−)KSR−K90), the cell growth rate was significantly decreased, which suggests that PVP may have a surfactant effect as well as a macromolecular crowding effect, as mentioned above. The reason behind IPKM cells not growing well in the serum-containing medium remains to be clarified. Although the addition of PVP K90 to the serum-containing medium may promote cell growth, it is easy to imagine that the cells would aggregate and adhere to the surface of the device. Unlike adherent cell cultures, the growth of cells in the culture proceeded from a lag phase, following seeding, to the log phase, where the cells proliferated exponentially. In this culture system (500 mL spinner flask), cell growth reached a plateau phase around day 40 because the medium volume was limited to 300 mL. These cells could be further expanded by transferring them to larger flasks and continuing subculturing. Unfortunately, we could not achieve a single-cell suspension culture in the present study. However, by slowly adapting the cells to the culture environment over a long period and selecting cells adapted to single-cell culture, it may be possible to obtain cells that grow as single cells that are not attached to a substrate [36]. As with the adherent culture, the growth efficiency of suspension cultures of IPKM cells may be further improved by optimizing the culture conditions.

In the future, we plan to perform ASFV infection experiments using the serum-free culture systems developed in this study, and the results are awaited.

## 5. Conclusions

We developed a serum-free culture medium for IPKM cells based on a KSR substitute for FBS. In the adherent cell culture system, the addition of cytokines, such as pCSF1 and pCSF2, as well as 2% PVP K90, is essential. In the suspension culture system, a serum-free medium modified to be Ca-free is necessary to prevent cell adhesion to the vessel. These serum-free media allow for the long-term adherent subculture of IPKM cells, as well as the suspension culture in spinner flasks, while maintaining their macrophage characteristics.

## Figures and Tables

**Table 1:** Media components.

Composition	FBSstd	DKSR + K90	αKSR + K90	Ca(−)KSR + K90
Medium	DMEM	DMEM	αMEM	Ca(−)DMEM
Insulin	10 μg/mL	–	–	–
FBS	10%	–	–	–
MTG	25 μM	25 μM	25 μM	25 μM
Anti-Anti	1%	1%	1%	1%
KSR	–	15%	15%	20%
pCSF1	–	0.25%	0.25%	0.25%
pCSF2	–	0.25%	0.25%	0.25%
PVP K-90	–	2% (*w*/*v*)	2% (*w*/*v*)	2% (*w*/*v*)
GlutaMAX	–	–	–	1%
Pyruvate	–	–	–	1 mM
Nucleosides	–	–	–	1%

The details of each component are described in the main text.

**Table 2:** Ratio of normal chromosomes after 3 months of subculture.

Media	Total Passage No.	No. (%) of 2n Chromosomes		
		<38	38	38<
FBSstd	P22	14 (28.6)	32 (65.3)	3 (6.1)
αKSR + K90	P21	16 (28.6)	36 (64.3)	4 (7.1)

## Data Availability

The data presented in this study are available on request from the corresponding author.

## Supplementary Materials

The following supporting information can be downloaded at: https://www.mdpi.com/article/10.3390/ani15040558/s1, Supplemental Table S1. PCR primers for cDNA cloning; Supplemental Table S2. PCR primers for semiquantitative RT-PCR.
