# Supplementary material for: Development of Serum-Free Culture Systems for an Immortalized Porcine Kidney-Derived Macrophage Cell Line
Source: Animals (Basel). 2025 Feb 14;15(4):558. doi: 10.3390/ani15040558 (PMC11851356; doi:10.3390/ani15040558)
Supplement: Supplementary file 1 [file animals-15-00558-s001.zip › animals-3451760-supplementary.pdf]

Table S1. PCR primers for cDNA cloning.

Gene (product size, bp)	Primer (F, forward; R, reverse)
(1st PCR)	
CSF1 (943)	F: 5′-AAAGTGAAAGTTTGCCTCGGTGCTCTTGGC-3′<br>R: 5′-TAGAATTCCCTTTACACTGGCAATTCCACC-3′
CSF2 (513)	F: 5′-TCTTTTGCTAGTGAGCCCAGTGCTCAGAG-3′<br>R: 5′-TGGCTTCTGGCTGCTGCAGGCTGCTTTAC-3′
(2nd PCR)	
CSF1 (799)	F: 5′-ACGAATTCGCCACCATGACCGCGCCGGGCG-3′<br>R: 5′-TAGAATTCCCTTTACACTGGCAATTCCACC-3′
CSF2 (456)	F: 5′-AGAATTCGCCACCATGTGGCTGCAGAACCT-3′<br>R: 5′-TGAATTCTTACTTTTTGACTGGCCCCCAGC-3′

The *Eco*RI recognition sites are underlined.

Table S2. PCR primers for semiquantitative RT-PCR.

Gene (product size, bp)*	Accession no.	Primer (F, forward; R, reverse) *
CSF1 (323)	NM_001244523.1	F: 5′-AAAGTGAAAGTTTGCCTCGGTGCTCTTGGC-3′<br>R: 5′-TAGAATTCCCTTTACACTGGCAATTCCACC-3′
CSF1R (354)	XM_003124100.6	F: 5′-AAGATTGGGGACTTTGGTCTGGCAAG-3′<br>R: 5′-AAGGAGGGAGCAGATCTGCTGGAAGG-3′
CSF2 (343)	NM_214118.2	F: 5′-AGCATGTGGATGCCATCAAAGAAGCC-3′<br>R: 5′-ACTTTTTGACTGGCCCCCAGCAGTC-3′
CSF2R (379)	XM_001924779.5	F: 5′-AGTTTGAGGTGGTCTACAGGCGGCTTC-3′<br>R: 5′-TCCTCCTTCTGCACTGGGGAACATTC-3′
IL-4 (310)	NM_214123.1	F: 5′-ACCCTGGTCTGCTTACTGGCATGTAC-3′<br>R: 5′-TCTTCTTGGCTTCATGCACAGAACAGG-3′
IL-4R (382)	NM_214340	F: 5′-ATCTGCCTATCCGACTATGTCAGCAC-3′<br>R: 5′-AGGTGAGTTCCGAGTACAGGTAGCTC-3′
LIF (278)	NM_214402.2	F: 5′-TGTCACAGCAACCTCATGAACCAG -3′<br>R: 5′-TTCACAGCACCAGGATTGAGGCTC -3′
GP130 (340)	EF151500.1	F: 5′-AGATAGAGCCGTCTCATACTCATGG-3′<br>R: 5′-TCATTTGGAGCAGTCCATTCTACCC-3′

* Primers were designed to flank intron(s).
